# Iodine

**Published:** 1863-12

**Authors:** H. A. Smith


DENTAL REGISTER OF THE WEST.

VOL. XVII.] DECEMBER, 1863. [No. 12.

### Original Essays and Communications.

### IODINE.

Read before the Cincinnati Dental Society—By H. A. SMITH, D. D. S.

There is a general tendency in those who practice a specialty, to give an undue importance to the deseases of the organ or set of organs to which they have especially given attention.

Whether a part or organ of the body be in a healthy or diseased condition, it can not be separately considered. If a tooth is presented for consideration, we should remember that it could not exist but as one of the many organs which go to make up the whole body; and that the condition of perfect health in the tooth is dependent upon a like condition in all the other organs, or more especially upon the condition of the organs in sympathy with or situated near to it. A tooth may become diseased from a disturbed condition of some organ in its vicinity; or a tooth already diseased may cause, through sympathy, similar affections in distant parts of the organism, even to a degree to disturb the general health.

We, as practitioners of dental surgery, seem practically to ignore these principles, or rather the fact that the various organs are mutually dependent upon each other. We are too much accustomed to regard local symptoms without a thought as to the general condition of the system,—when, in fact, the local conditions are often dependent upon the constitutional state.

If it is true, that we restrict ourselves, in the diagnosis of cases coming within our province as dentists, to local symptoms almost entirely, it follows that the remedies which we mostly use in their treatment are topical or local in their application. And it is true that we find these the most efficient.

The number and variety of these remedies are quite limited; and we are, of necessity, restricted in the use of therapeutic agents. For this reason, it is important that dentists should become familiar with the therapeutic operation of each of the remedies which he uses; and this can only be done by watching the effects of the medicine, after first noting carefully the pathological condition of the parts diseased.

Iodine has been used as a medicine, both internally and locally, for many years. But its value as a remedy in the several forms of disease which come strictly within the province of the dentist, has not been known, or, at least, not appreciated, until within a very few years.

One of the most common forms of disease for which iodine is used locally by the dental practitioner, is *periostitis*, or inflammation of the "intra-alveolar periosteum."

Without going fully into a pathological description of this abnormal condition, it is necessary, to a proper understanding of the subject, that we should refer, incidentally, to the condition of the parts involved in disease.

There are several forms in which this disease may present itself for treatment, dependent upon the cause that has induced it, or upon the length of time which has elapsed since diseased action was set up.

The condition in which a cure is most likely to be effected, is in the early stage of the disease, where we find simply a congested state of the periosteal membrane, with the vessels relaxed. A certain amount of blood is only required for the healthy condition of this membrane. We find here an excess; and an undue amount of blood can not be retained in a part, without a tendency to diminish the quantity elsewhere.

Now, depletion by local blood-letting would naturally suggest itself as the proper treatment. It is often efficacious; but we do not so much desire to diminish the quantity of blood as to restore its equilibrium. For this purpose, we resort to a *counter-irritant;* and by applying it in near contiguity, we direct the blood from the congested tissue, and at once aid in bringing about a healthy reaction.

In the more advanced or chronic form of periostitis, or those cases where inflammation has set up but has not proceeded to suppuration, we may expect decided results from the local use of iodine as a counter-irritant. It appears to act as a *revulsive*, by diverting the disease from the part simply by its irritating effect upon the surface. This principle is employed largely in the cure of disease. In these cases, where we have a grade of inflammation stopping short of suppuration, it is important that the counter-irritant act intensely rather than extensively; and for this reason, the revulsive or irritant impression should be brought as near the seat of disease as possible. In the first form of periostitis that I mentioned, where the vessels are simply congested, it is possible to induce inflammation of a higher grade, by a strong impression upon the surface over the part. This would hardly occur with the use of iodine, unless used in its concentrated form, or too often applied.

Owing to a peculiar *diathesis* or condition of the system, the mucous membrane of the mouth of some patients is more readily effected by the irritant properties of iodine than in others. Ordinarily in cases of congestion, it is well not to produce any organic change of the surface membrane. But if inflammation has set up, threatening suppuration, a decided impression must be produced. The use of the iodine should be continued until a deep brown stain of the surface is effected, followed, possibly, by the desquamation of the mucous membrane.

If the disease has not been arrested, the periosteum is subject to the several stages of inflammation; the suppurative process is set up, pus is formed, and abscess is the result. The pulp of the tooth is now dead, or in a rapid state of disorganization. And if the pus has not made an opening through the alveolus for its passage, free opening should be had through the fang, so that the pus may escape, and in order that the iodine may be made to pass through into the ulcer.

The use of iodine in these cases is frequently attended with good results. The modus operandi of the remedy, however, is, at best, only conjectural. That its action is in part chemical, is probable. By its affinity for some one or more constituent of the morbid secretion, neutralizing it and thus rendering it less noxious to the tissues in contact, and also rendering it more easy of elimination and absorption by the processes of nature.

The *alterative* properties of iodine are well attested, and it may be that its efficiency in these cases may be traced to its alterative effects. The nature of the action or change produced by an alterative remedy is never fully understood. But this is no reason why we should not conjecture its probable action; for possibly we may be correct, or at least institute an inquiry as to the truth.

We have here, then, an abnormal condition of the tissues. The alveolus is absorbed or excoriated by the chemical solvent power of the effused fluids. In its place we have pus.

It is in these cases of perverted action that iodine may be used beneficially. Besides acting chemically upon the secretions, as I have already referred to, it probably acts in a similar way in modifying the tissues already reduced to a low grade of vitality and changed in their organic constituents. This morbid or abnormal state of the tissues causes them to be much more readily influenced by agents tending to their destruction, than healthy tissue whose vital power is much more vigorous. The over-stimulation which is produced by the use of iodine, changes the ultimate organic constituents of the abnormal tissues—causing them to be broken up and disappear; at the same time, the perverted action in the parts is corrected, and soon new and healthy tissue is formed, taking the place of the old. It is somewhat in this manner that the efficiency of iodine as an alterative may probably be explained.

Iodine, when applied directly to the serous membrane in an inflamed condition, as in injecting the cavity of the antrum, has a tendency to diminish the amount of secretions by its astringent properties. It is probable that it also acts chemically, and renders the morbid secretion less acrid, and thus diminishes inflammatory action in the parts.

As a local application in those cases of acute neuralgic pains, when apparently confined to the maxillary branches of the trifacial nerve, I have found its effects most happy. There is, in these cases, a concentration or an excessive amount of nervous action, and consequently a diminution in the parts surrounding,—just as I remarked in relation to the tendency of the blood to diminish in amount in other parts of the body, if we have it directed strongly to a particular locality. For the same reasons that we apply a counterirritant, iodine, to diminish the congested state of the blood-vessels in a part, we would apply it to diminish an excessive amount of nervous sensibility. The iodine acting as a revulsive by irritating the surfaces in near proximity, the disease is diverted from the part.

In those more complicated forms of disease, involving both the hard and soft tissues of the mouth to a much greater extent than in periostitis, iodine or some of its compounds is now extensively used. Constitutional treatment in these cases, especially where they are the result of a syphilitic virus in the system, can not be dispensed with.

The effect of iodine used locally in these cases is no doubt very similar to that which it produces in diseased conditions already described. In the treatment of this form of disease, we have not been consulted to any extent; and for this reason it would not be in the province of this paper to discuss it more at length.

